# A comprehensive hybridization model allows whole HERV transcriptome profiling using high density microarray

**DOI:** 10.1186/s12864-017-3669-7

**Published:** 2017-04-08

**Authors:** Jérémie Becker, Philippe Pérot, Valérie Cheynet, Guy Oriol, Nathalie Mugnier, Marine Mommert, Olivier Tabone, Julien Textoris, Jean-Baptiste Veyrieras, François Mallet

**Affiliations:** 1 Joint research unit, Hospice Civils de Lyon, bioMerieux, Centre Hospitalier Lyon Sud, 165 Chemin du Grand Revoyet, 69310 Pierre-Benite, France; 2 Bioinformatics Research Department, bioMerieux, 376 Chemin de l’Orme, 69280 Marcy l’Etoile, France; 3 EA 7426 Pathophysiology of Injury-induced Immunosuppression, University of Lyon1-Hospices Civils de Lyon-bioMérieux, Hôpital Edouard Herriot, 5 Place d’Arsonval, 69437 Lyon Cedex 3, France

**Keywords:** Transcriptomics, Biostatistics, Microarray, Repetitive elements

## Abstract

**Background:**

Human endogenous retroviruses (HERVs) have received much attention for their implications in the etiology of many human diseases and their profound effect on evolution. Notably, recent studies have highlighted associations between HERVs expression and cancers (Yu et al., Int J Mol Med 32, 2013), autoimmunity (Balada et al., Int Rev Immunol 29:351–370, 2010) and neurological (Christensen, J Neuroimmune Pharmacol 5:326–335, 2010) conditions. Their repetitive nature makes their study particularly challenging, where expression studies have largely focused on individual loci (De Parseval et al., J Virol 77:10414–10422, 2003) or general trends within families (Forsman et al., J Virol Methods 129:16–30, 2005; Seifarth et al., J Virol 79:341–352, 2005; Pichon et al., Nucleic Acids Res 34:e46, 2006).

**Methods:**

To refine our understanding of HERVs activity, we introduce here a new microarray, HERV-V3. This work was made possible by the careful detection and annotation of genomic HERV/MaLR sequences as well as the development of a new hybridization model, allowing the optimization of probe performances and the control of cross-reactions.﻿﻿﻿

**Results:**

HERV-V3 offers an almost complete coverage of HERVs and their ancestors (mammalian apparent LTR-retrotransposons, MaLRs) at the locus level along with four other repertoires (active LINE-1 elements, lncRNA, a selection of 1559 human genes and common infectious viruses). We demonstrate that HERV-V3 analytical performances are comparable with commercial Affymetrix arrays, and that for a selection of tissue/pathological specific loci, the patterns of expression measured on HERV-V3 is consistent with those reported in the literature.

**Conclusions:**

Given its large HERVs/MaLRs coverage and additional repertoires, HERV-V3 opens the door to multiple applications such as enhancers and alternative promoters identification, biomarkers identification as well as the characterization of genes and HERVs/MaLRs modulation caused by viral infection.

**Electronic supplementary material:**

The online version of this article (doi:10.1186/s12864-017-3669-7) contains supplementary material, which is available to authorized users.

## Background

The recent sequencing of model organisms unveiled the large proportion of repetitive elements (REs) in many species. In human, it is estimated that half of the genome is populated by REs and that retrovirus-like sequences amount for 8% of its coverage [1]. HERVs and MaLRs elements are organized into multi-copy families, for each of which, tens to thousands of distinct loci are scattered throughout the human genome, representing a pool of approximately 200,000 individual HERV loci. While bioinformatics approaches identified 103 HERV families and 1 MaLR family [1], only 40 HERV families were characterized in wet-lab studies [2–4]. Part of this genomic heritage is thought to originate from ancestral and independent retroviral infections within the germ line, before reinfection, retro-transposition and error-prone amplification steps during the evolution, leading to the formation of multi-copy families [5]. To date, no infectious endogenous virus has been detected in human, however 30% of the whole retrovirome is estimated to have a transcriptional activity [6]. Multiple functions have been assigned to these elements: HERVs have been demonstrated to act as canonical and alternative transcription start sites [7] (up to 30% of human and mouse TSSs are located in REs [8]), transcription termination sites [9] as well as splice donor and splice acceptor sites [10]. REs have further been suggested to be instrumental in the long intergenic non-coding RNA (lincRNA) regulatory system, where a majority of lincRNAs have been found to contain REs [11]. HERVs are increasingly associated with distinct physiological and pathological processes. One notable example is provided by the two syncytins genes that have been co-opted in human (and other mammals) to mediate placentation [12]. More recently, HERV-H loci have been shown to be instrumental in the maintenance of pluripotency [13]. Other investigations have further described associations between HERVs reactivation and multiple sclerosis [14–16], solid [17, 18] and hematological [19] tumors. Taken together, these studies show that REs provide binding sites for mammalian TFs and that they have rewired a number of developmental regulatory networks.

The central issue in the study of the HERV transcriptome arises from the phylogenetic proximity among the elements of a given HERV family, making the measure of each transcript technically challenging. Initially, RT-PCR techniques combined with degenerate primers [20] and low-density microarrays [18, 21] were developed to measure trends within families without, however, providing locus-specific information. Expressed sequence tags (ESTs) approaches gave a more comprehensive view of the HERV transcriptome but failed in many instances to identify the exact genomic source of expression [22]. Recent initiatives took advantage of probes targeting repetitive elements in commercial microarrays to monitor HERV behavior where, in addition to restricting their analysis to a small number of probes, the specificity of the probes was not evaluated [23]. More recently, HERVs transcription was also measured in various contexts using next generation sequencing (NGS) [24], which, while promising, remains difficult due to the ambiguity in assigning short reads mapping to more than one genomic location. For instance, in a study of HML-2 elements in teratocarcinoma cell line, Bhardwaj et al. showed that 47% of their reads had multiple alignments [25]. Two elegant initiatives sought to address this limitation by either using host surrounding sequences to anchor HERV copies [26] or by assigning multi-mapping reads probabilistically to specific locus based on the local genomic tag context [27]. However, in addition to assume that HERVs flanking regions are expressed, these approaches can probably not resolve multi-mapped reads for more than few hundred bases at the edges of HERV copies, leaving the ambiguity unchanged in the central regions.

Because HERV expression is globally low [28], very deep sequencing is required to capture the diversity of HERV transcripts among the many other and more abundant human transcripts, making unbiased NGS experiments costly and ineffective in this context. Targeted sequencing could alternatively be considered to reduce the experimental burden by specifically amplifying the transcripts of interest, as is typically applied in 16S metagenomic sequencing. This type of approach could either be performed at the family or locus level. The design of family-specific degenerate primers or locus-specific primers would however require an elaborate step of primer selection ensuring both family/locus specificity (as illustrated in Pichon et al. for PCR amplification of the Pol region [18]) and compatible annealing temperature for unbiased quantification. To our knowledge, no such systematic targeted sequencing approach has been proposed so far. The work presented in this study applies such methodology on microarray using a probe selection pipeline that aims to both maximize probe efficiency and mitigate non-specific reactions, minimizing thus the analysis step for the end-user. Microarrays platforms and in particular Affymetrix instruments are now deployed in many research laboratories and the cost per experiment makes microarrays affordable compared to NGS, with a reduced time-to-result.

Two custom microarrays were previously designed in the laboratory based on a unicity criterion and a specificity score. The first meant that only candidate probes with a single perfect match were selected [29], whereas the second estimated a cross-hybridization risk using the nature and position of mispairing (mismatches, MMs and gaps) in probe-target hybrids [13]. Training sets consisting of PM and MM probes were introduced on both arrays to evaluate and refine these strategies of cross-hybridization control. Both platforms allowed the identification of cancer-specific loci (testis [29], prostate [13, 30], colon [13] subsequently validated by qRT-PCR on a large cohort [31]) and the assignment of LTR functions [13, 29], but did not prevent cross-reactions to occur, raising the need for an improved approach.

Building on these two experiences and leveraging the high-density Affymetrix format (5 micron feature size), we introduce here a new platform HERV-V3 which, like the previous versions, aims at measuring HERVs at the locus level. The two main improvements lie in the almost complete coverage of HERVs and their ancestors as well as the introduction of a specificity criterion based on a new hybridization model, named hereafter, the Pentamer rEgion-dependent Hybridization Model (PEHM). The aim of this model is to predict the affinity of any probe-target hybrid, and therefore, to evaluate the potential of cross-hybridization by determining whether a probe of interest hybridizes only with its target. Along HERVs elements, five additional repertoires were introduced on HERV-V3 that fall in three categories, repetitive elements (MaLRs and active LINE-1 elements), non-repetitive elements (lncRNA and a selection of 1559 human genes) and common infectious viruses. While the array design is primarily aimed at identifying HERVs and MaLRs implicated in physiological and pathological processes, broader applications can be envisioned with these repertoires, such as the detection of virus replication along with the monitoring of HERVs/MaLRs and genes modulation. In the following, we successively (i) describe the main steps of the array design, (ii) compare our probesets with those of Affymetrix on 1559 common genes according to the MAQC criteria and (iii) demonstrate that for a selection of loci characterized as tissue/pathology specific, the pattern of expression observed on HERV-V3 is consistent, illustrating the relevance of such platform as research tool.

## Methods

The design of the HERV-V3 array followed three main steps: (i) the genomic detection and the annotation of HERVs/MaLRs elements presented here, (ii) the development of a hybridization model to prevent cross-reactions and (iii) the design of the probes. The hybridization model was fitted on the HERV-V2 training set, made of degenerated Affymetrix probesets (see below).

### Database creation

**Fig. 1:**
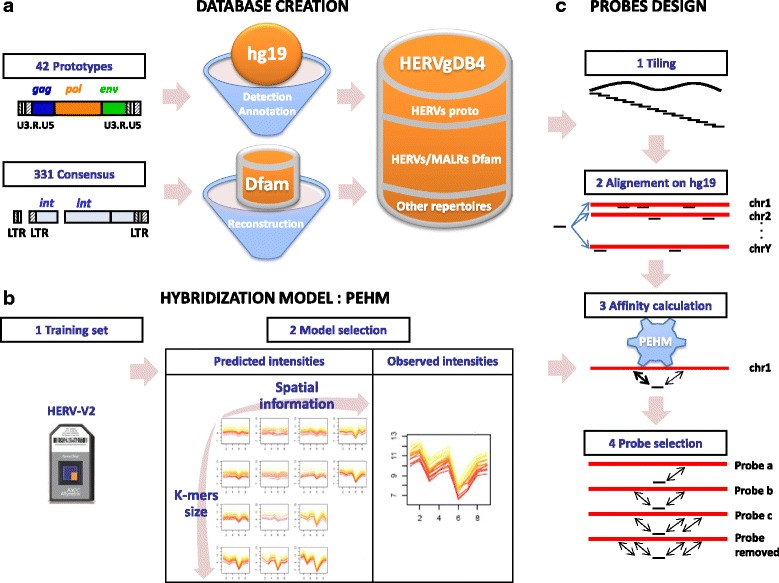
Mains steps of the HERV-V3 array design. The design involved three steps of (**a**) database creation, where HERV copies were either detected by RepeatMasker using 42 prototypes or reconstructed from Dfam predictions; (**b**) development of a hybridization model, illustrated by models predictions and observed intensities on Affymetrix probeset associated with CD59 gene; and (**c**) design of probes and probesets. The difference of annotation level between consensus and prototypes is shown, where LTR subregions and ORFs are only identified in prototypes. It can further be noted that the agreement between observed and predicted intensities increases with the k-mers size and the complexity of spatial information (a more thorough description is provided in the Additional file 3: Figure S1)

The HERV-V3 array ambitions both to cover the whole human retrovirome and provides functional annotations when possible. These annotations are primarily meant to address hypotheses on LTRs functions (i.e. promoter or polyA) and to support data interpretation at the level of gag/pol/env regions and their putative ORFs. A first step of genomic detection and annotation was performed (Fig. 1a), step which is non-trivial given that HERV classification remains incomplete [32]. To this end, two different sources of information were used, a set of prototypes associated with 42 families described in the literature [3, 21, 33, 34] for which annotations were generated in the laboratory (Additional file 1: Supplementary Notes, section 1, and Additional file 2: Table S1), and 331 Repbase consensus for which no annotation could easily be generated [35]. In the first case, prototypes were aligned on the human genome (hg19) using RepeatMasker, leading to a set of annotated HERVs called hereafter “HERVs prototypes”. In the second case, fragmented HERV elements were retrieved from Dfam, a database of repetitive elements detected by RepBase consensus [36], and subsequently reconstructed (Cf Additional file 1: Supplementary Notes, section 2). This two levels strategy was devised to generate accurate annotations on elements detected by prototypes and to recover as many HERVs as possible using the representativeness of Repbase consensus. All the detected and annotated elements were finally stored in a database named hereafter HERVgDB4.

### Hybridization model

Once the database created, a hybridization model, PEHM, was developed to predict the probe cross-hybridization potential (see Fig. 1b). This was made possible by an explicit modeling of MMs and gaps, allowing thus a precise quantification of mispairing. Hybridization models have been explored in the past decade, where the focus was more on modeling perfect match hybridization to improve microarray design, data interpretation [37–39] and to detect cross-reactions [40]. Here, the goal of the model is to predict the affinity of DNA hybrids with possible MMs and gaps, from which the cross-reaction potential can be deduced. The model we introduce, PEHM, is along the same lines as Li & Wong and Zhang models [40, 41] that express the probe intensity as a product of the affinity for its target with the target concentration (otherwise called expression measure):

1 $$ {I}_{i j}={\theta}_i.{\varphi}_j+{\varepsilon}_{i j};{\varepsilon}_{i j}\sim N\left(0,\ {\sigma}^2\right),{\displaystyle \sum_n}{\theta}_n^2= N $$

with *I*_*ij*_ the intensity of probe j on array i, *φ*_*j*_ the affinity of probe j, *θ*_*i*_ the expression level of the gene targeted by probe j on array i and *ε*_*ij*_, an independent identically distributed error term centered on 0. Because of the product between affinity and target concentration, a constraint is required to allow parameter identifiability. An important difference between PEHM and Li & Wong is that the parameter of interest is the affinity in the first case, while it is the target concentration in the second. Consequently, instead of considering affinity as a nuisance parameter and imposing the identifiability constraint onto it [41, 42], the constraint here is imposed on the RNA quantity, where the sum squares of *θ*_*n*_ is set to N. Furthermore, PEHM links the probe-target affinity to the DNA sequence by modeling the affinity as a sum of k-mers effects, similarly to Zhang et al. This initial model is then extended in four ways: (i) given that DNA structural properties (i.e. flexibility, stability) depend on the interactions between neighboring base pairs, pentamers instead of dimers were used to improve affinity modeling (data not showed). (ii) While the spatial effect was previously modeled through position weights (modulating k-mers in function of their position, Zhang et al.) or by estimating k-mers at each position of the probe [37], an approximation of the latter is chosen here by considering three sub-regions of identical size in the probes. Although less precise, this approximation reduce by a factor 7 the number of parameters in comparison with Mei et al. approach. (iii) MM and gap 5-mers are taken into account as well as (iv) interactions between mismatches, following the idea that the k-mers additivity breaks down in presence of multiple MMs [43]. Overall, the affinity is expressed as follows:

2 $$ {\varphi}_j={\displaystyle \sum_l}{\displaystyle \sum_k}{\beta}_k^l{X}_{j k}^l+{\displaystyle \sum_m}{\delta}_m{Z}_{j m} $$

With β_k_^l^ the coefficient associated with k-mer k in sub-region l, X_jk_^l^, the indicator matrix providing the number of k-mer k in region l of probe j, δ_m_ the coefficient associated with interaction m and Z_jm_ the indicator matrix providing the presence or absence of interaction *m* in probe *j*. Although conceptually straightforward, the use of MM and gap 5-mers dramatically increased the number of parameters from 1024 to 113,664. Model parameters were estimated using the LASSO shrinkage method [44] to prevent overfitting and consequently improve the model predictions. The model training was performed in 10-folds cross-validation on the HERV-V2 training set that consists of 20 probesets derived from the Affymetrix U133 array. Each probeset contains the 10 original U133 PM probes along with 1800 degenerated MM/Gap probes including single, double MMs and single gaps, which represent a total of 37,200 probes. The data used in the model training arose from 36 microarray experiments performed on healthy and tumor tissues (colon, breast, ovary, uterus, prostate, testis, lung and placenta) carried out in a previous study [6]. Once the model defined, an “hybridization threshold” was determined on the affinity to distinguish stable from unstable hybrids in the probe design. This threshold was set such that 90% of the probes with an affinity under this threshold have intensity under the background noise. The model performances are illustrated on Additional file 3: Figure S1 (enlarged version of Fig. 1b) using Affymetrix probeset associated with CD59 gene.

### Probes and probesets design

PEHM was used in the array design to select probes that are both specific and thermodynamically efficient. To do so, the number of hybridizing targets (specific and cross-hybridizing) was predicted for each candidate probe by PEHM, and only probes capable of hybridizing with one to three targets were retained. The array design involved three steps of tiling, probe selection and probeset generation (see Fig. 1c). Each region of interest was tiled into 25 bp candidate probes with a step size between 1 and 4 bp depending on the perimeter coverage and the quality of its annotation. For instance, a step of 1 bp was used for HERVs prototypes to ensure that all candidate probes were considered in this relatively small and well annotated perimeter. For each candidate probe, the affinity with its specific target was then computed to assess its thermodynamic performance. If the affinity exceeded the hybridization threshold, the probe was subsequently aligned against a reference library using BWA [45]. Two libraries were generated covering either the repetitive fraction of the genome (hg19 regions masked by RepeatMasker) or its complementary. The advantage of dividing the genome in two partitions was to reduce substantially the execution time of BWA whose complexity is in l. n^0.628^. m (l the number of probes, n the size of the reference library and m the probe size). Affinities were then calculated with PEHM for each hits, from which probes were classified into three categories: “specific”, if only one hit was above the hybridization threshold, “potentially cross-hybridizing”, if less than four hits exceeded the hybridization threshold and “non-specific” otherwise. In this latter case, the candidate probes were excluded. This relatively permissive strategy was designed to include as many loci as possible on HERV-V3, even those part of the most highly repetitive families. Also, given that a small proportion of HERV loci is generally expressed in a given biological context, the probability that two cross-hybridizing transcripts are simultaneously expressed is reduced.

In Mei et al., the generation of Affymetrix probesets was based on a score that maximizes probes responsiveness (quantity related to affinity), probes uniqueness (specificity) and inter-probes distance (spreadness) [37]. In HERV-V3 design, the affinity and specificity were controlled at the probe selection step, while the probeset size, the spreadness, and cross-reaction criteria were taken into account in the probeset generation step. More specifically, a probeset was required to contain between 3 and 6 probes to yield a robust estimation of gene-expression while keeping the probeset size low due to the large number of targeted elements. This relatively small lower bound was motivated by the high level of homology existing in certain families, preventing the definition of larger probesets. We therefore preferred smaller probesets than missing out loci. This point is further discussed in the evaluation of the platform performances. A probeset was restricted to a 400 bp region, in which, a maximum 30% overlap between two neighboring probes was allowed. This means that if two probes separated by less than 8 bp pass the specificity test described above, only one will be kept in the final probeset. Cross-hybridization was also mitigated at the probeset level where for a given probeset, cross-hybridizing probes had to cross-react with distinct loci and at least one probe had to be specific (with no cross-reaction). Approximately 2 weeks were necessary to run this three steps probe definition pipeline on a server (16 CPU, 128 GB of RAM).

### RNA sources and ethical considerations

The technical performances were evaluated on the MAQC samples, composed of two independent samples (A, Stratagene Universal RNA, and B, Ambion Human Brain RNA) from which two titration samples were generated (C and D, consisting of 3:1 and 1:3 ratios of A to B, respectively). Each sample was performed in technical triplicate. The biological validation was, on the other hand, performed on three different tissues (colon, placenta and prostate) and two primary human cell lines (OSCAR and EBJ14). The colon (tumor and adjacent normal tissues in two patients) and placenta RNA samples were purchased from Clinisciences and Ambion.

The prostate samples were isolated from post-surgery (radical prostatectomy) prostate cancer and prostate normal tissue, then treated by micro-dissection. Post-surgery prostate sample were provided by the Tumorothèque du Centre Hospitalier Lyon-Sud (Pierre Benite, France). The tissue samples conservation after prostate surgery in Centre Hospitalier Lyon-Sud was performed with the local ethics committee approval (Comité de Protection des Personnes de Lyon). All patients were informed through an individual notice during the hospital admission and then gave their verbal consent, as required by the French Loi de Bioéthique (2004), for the sample conservation and research use. Prostate RNAs were extracted following the Trizol protocol (Invitrogen) and purified on Rneasy columns (Qiagen). The quality of all RNA samples was assessed with the Bioanalyser 2100 capillary.

RNA extracted from the OSCAR and EBJ14 primary human cell lines were provided by the Brain Research Institute (INSERM U846, Université Lyon 1, Lyon, France). OSCAR cells consist of human embryonic stem cells (hESCs) cultured through the addition of FGF2 in the culture medium. EBJ14 (embryoid bodies) cells were obtained by culturing the OSCAR cells in non-adherent culture dishes without FGF2, environment in which cells form floating structures that spontaneously differentiate [46].

### RNA amplification and labeling

The cDNA synthesis and amplification steps were performed from 16 ng of RNA using the Ovation Pico WTA System V2 kit (Nugen). Briefly, a first strand cDNA was generated from total RNA using a mixture of random and polydT DNA/RNA chimeric primers, followed by the synthesis of the complementary strand. The mRNA strand within the cDNA/mRNA complex was fragmented in order to create priming site to permit the DNA polymerase to synthesize the second cDNA strand. The double-stranded cDNA with a short DNA/RNA heteroduplex was amplified using the strand displacement based Single Primer Isothermal Amplification (SPIA) method. Schematically, RNase-H removed the RNA portion of the heteroduplex sequence and revealed a site for binding the DNA/RNA chimeric SPIA primer. DNA polymerase synthesized a new cDNA starting at the 3′ end of the primer, displacing the existing forward strand released as ssDNA. Priming with the chimeric SPIA primer recapitulated the heteroduplex creating a new substrate for RNase-H and the initiation of the next round of cDNA synthesis and ssDNA release.

The resulting amplified ssDNA was purified using the QIAquick purification kit (Qiagen), from which, total DNA concentration was measured using the NanoDrop 1000 spectrophotometer (Thermo Scientific) and the product quality was checked on the Bioanalyser 2100. Five micrograms of purified ssDNA were fragmented and labeled with the Encore Biotin Module kit (Nugen): the cDNA products were fragmented by enzymatic process into 50–100 bp fragments and subsequently labeled via enzymatic attachment of a biotin-labeled nucleotide to the 3-hydroxyl end of the fragmented cDNA. The resulting target was mixed with standard hybridization controls and B2 oligonucleotides following the recommendations of the supplier. The hybridization cocktail was heat-denatured at 95 C for 2 min, incubated at 50 C for 5 min and centrifuged at 16,000 g for 5 min to pellet the residual salts. The HERV microarrays were pre-hybridized with 200 μ*L* of hybridization buffer and placed under stirring (60 rpm) in an oven at 50 C for 10 min. The hybridization buffer was then replaced by the denatured hybridization cocktail. Hybridization was performed at 50 °C for 18 h in the oven under constant stirring (60 rpm). Washing and staining were carried out according to the protocol supplied by the manufacturer, using a fluidic station (GeneChip fluidic station 450, Affymetrix). The arrays were finally scanned using a fluorometric scanner (GeneChip scanner 3000 7G, Affymetrix).

### Bioinformatics microarray analysis

Quality checks were systematically performed before microarray data analysis. The indicators examined were (i) the amplification and hybridization Affymetrix controls, (ii) the median absolute deviation versus the intensity median (MAD-Med) representation, (iii) the Normalized Unscaled Standard Error (NUSE) and (iv) the Relative Log Expression (RLE) [47].

Four pre-processing (background correction, normalization and summarization) approaches were compared, RMA [42], two alternatives to RMA and Li & Wong [41]. The two alternatives differ from RMA by their background correction step: the background noise is estimated either globally using the 15th percentiles of tryptophan probes or at the probe level using the median intensity of antigenomic probes with identical GC-content. The antigenomic probes have been introduced on exon arrays to estimate the non-specific hybridization effect related to probes GC content [48]. Their design is such that they do not match any location in the human genome and cover a wide range of GC content.

Lastly, the search for differentially expressed genes (DEG) was performed using LIMMA [49]. This method relies on a moderated t-stastistic, robust for small numbers of arrays. *Q*-value and fold-change thresholds of 0.01 and 2 respectively were used in the technical and biological validations. To ensure that probesets identified as differentially expressed were not in the background noise, a threshold of 2^4^ was set on the median of the technical replicates (*n* = 3), intensity for which CVs across technical replicates were under 15%.

## Results and discussion

### Database and microarray contents

A total of 29,859 and 169,821 HERV prototypes and HERVs Dfam were collected and stored in HERVgDB4 (see Table 1). Six additional repertoires were added to this database, (i) 228,429 MaLRs (ancestors of HERVs) retrieved from Dfam and processed in the same way as the HERVs Dfam; (ii) 192 centromeric HERV elements (absent from hg19) shown to be reactivated in HIV infection [50]; (iii) a selection of 1072 putative active LINE-1 elements arising from the union of L1Base and dbRIP databases [51, 52]; (iv) 3777 long non-coding RNAs coming from two studies [53, 54], cleared of repetitive sequences with RepeatMasker (total coverage = 366.8 Mb); (v) 289 infectious viruses and (vi) 1559 genes involved in eight pathways (immunity, inflammation, cancer, central nervous system affections, differentiation, telomere maintenance, chromatin structure and gag-like genes, see Additional file 4: Table S2). Each of those 1559 genes are targeted by three probesets, two originating from commercial Affymetrix arrays (U133 and HTA v2), and one from our design. Put another way, the expression level of any of these 1559 genes is simultaneously measured by a U133 and HTA probeset as well as a probeset designed using the PEHM model. Their relative performances, presented in the following sections, provide a simple way to validate our probe design. For simplicity, we will call these probesets gU133, gHTA, and gPEHM. To ensure that we can rely on gU133 and gHTA as internal controls, we checked whether gU133 show a similar behaviour on HERV-V3 and HG-U133 Plus 2.0 array. A large correlation (*R* ^2^ = 0.811, probeset level) was found on gU133 probetsets between the two arrays, supporting thus the use of gU133 and gHTA as standard for comparison (Additional file 5: Figure S2). Overall, HERV-V3 contains 372,976 elements, represented by 2.7 million probes. Probes were synthesized in sense and antisense (5.3 million in total) to accommodate with any amplification protocols and retain transcripts strand, given that some LTRs were shown to exhibit bidirectional promoter activity [55].

**Table 1:** Number of elements and functional sub-regions contained in HERVgDB4 (left) and designed on HERV-V3 (right) where one probeset is defined by sub-region

Repertoire	HERVgDB4 (database)		HERV-V3 (array)		
	Number of elements	Number o﻿f sub-regions	Number of elements	Number of probesets	Number of elements
HERV prototypes	29,859	90,106	29,807	45,374	29,859
HERV centromeric	192	589	24	29	192
HERV Dfam	169,821	342,482	154,535	283,641	169,821
MaLR Dfam	228,429	45,543	179,323	311,286	22,8429
LINE1	1072	4627	664	1416	1072
lncRNA	3812	3819	3777	3777	3812
Viruses	291	386	289	368	2044
gPEHM	1559	1559	1559	1559	8743
gU133	1559	NA	1559	3884	42,964
gHTA	1559	NA	1559	35,398	344,002
Affymetrix Controls	NA	NA	NA	177	20,895
Total	435,040	898,998	372,976	686,869	2,651,585

The discrepancy between the number of elements in the database and on the array is due to cross-hybridizing elements discarded during the design

### Platform evaluation

Following on the MAQC consortium, the technical performances of the platform were first studied based on repeatability and accuracy, which have become standard in platform evaluation [56]. Accuracy has commonly been assessed either by comparing the estimated dilution mixtures from array intensities to their theoretical values, or by computing the titration response. The former relies on the assumption that in a titration sample, the signal of a given transcript is a linear combination of the signals measured in the two original samples (*C* = *α*_C_ *A* + *β*_C_ *B* and *D* = *α*_*D*_ *A* + *β*_*D*_ *B*). If this assumption is satisfied, the fractions estimated on the array should be centered on the dilution mixtures *β*_C_ = 0.25 and *β*_*D*_ = 0.75. The latter measures the coherence between the abundance of the hybridized RNA and the intensity measured on the array using two samples A and B and their mixture C (75% A + 25% B) and D (25% A + 75% B). This titration implies that for any gene i, if the true expression level *A*_*i*_ > *B*_*i*_, then the average intensities across triplicates are expected to follow *A*_*i*_ > *C* _*i*_ > *D*_*i*_ > *B*_*i*_, and conversely, if *B*_*i*_ > *A*_*i*_, then *B*_*i*_ > *D* _*i*_ > *C*_*i*_ > *A*_*i*_.

**Fig. 2:**
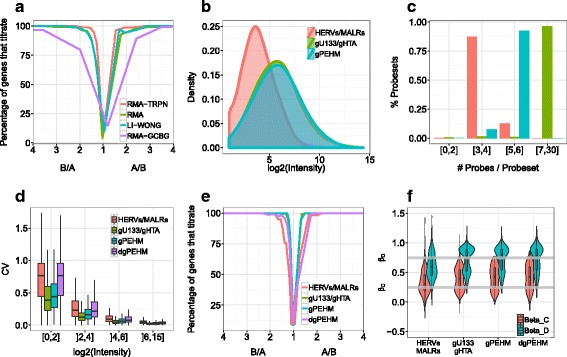
Platform evaluation. **a** Pre-processing methods were evaluated on the whole array using the titration response as a function of the fold-change between samples A and B. Probesets were binned according to the fold-change values between A and B. Unlike GCBG-RMA, the three methods RMA-TPRN, RMA and Li-Wong present narrow titration curves, indicative of good performances. The two confounding factors (**b**) intensity and (**c**, same colour code as in 2**b**) probeset size distribution are represented in HERVs/MaLRs, gU133/gHTA and gPEHM compartments: the intensities are lower in HERVs/MaLRs than in genes (gPEHM, gU133/gHTA), reffecting a smaller proportion of expressed loci in the former. The three compartments, HERVs/MaLRs, gU133/gHTA, gPEHM, and downsized gPEHM (dgPEHM) are compared on (**d**) repeatability (CV) and accuracy measured both by (**e**) the titration response and (**f**) the estimated dilution mixture (${\widehat{\beta}}_{\mathrm{C}},{\widehat{\beta}}_{\mathrm{D}}$). The grey horizontal lines in (**f**) symbolizes the theoretical mixture values *β*_C_ and *β*_D_. Only probesets differentially expressed between samples A and B (fold-change A/B and B/A > 2, *P* < 0.01) were used to generate the boxplots in (**f**). The gene repertoires show similar level of repeatability and accuracy (similar median CVs, titration curves and ${\widehat{\beta}}_{\mathrm{C}},{\widehat{\beta}}_{\mathrm{D}}$ distributions), whereas HERVs/MaLRs performances are slightly lower, due to smaller probesets

This quantity was first utilized to evaluate normalization procedures. Four methods were tested, Li-Wong [41], RMA [42] and two alternatives, RMA-TRPN and RMA-GCBG, that differed by their background correction (see the Bioinformatics microarray analysis section). The methods gave similar performances except RMA-GCBG whose titration curve showed a broader spread (see Fig. 2a). Inter-methods differences were quantified by measuring the *B*_*i*_/*A*_*i*_ ratio at which 75% of the probesets show a monotonic titration. This ratio was reached at 1.45, 1.53, 1.6, 2.19 in RMA-TRPN, Li-Wong, RMA and RMA-GCBG, which prompted us to keep RMA-TRPN in the following. In theory, PEHM could also be used for data pre-processing. However, because affinities are likely to be inferred more accurately by direct data estimation (RMA) than sequence based prediction (PEHM) and because RMA has received a large consensus in the community [57], we chose RMA for normalizing our data in this study.

We then compared our probe design with Affymetrix’s approach and checked whether the quality of measure was equivalent across repertoires (genes versus REs). The repeatability and the titration response were compared across the HERVs/MaLRs, gPEHM and gU133/gHTA compartments. Because the first two repertoires target two different sets of genomic elements while deriving from the same design method, their comparison reveals how our design approach performs on cellular genes and repetitive elements. The last two, on the other hand, target the same genes while deriving from two distinct design methods. Their comparison sheds light on the relative performances between Affymetrix design method and ours. Since gPEHM and gU133/gHTA gene repertoires presented higher intensity and larger probeset size (10 and 5.8 probes/probeset on average in gU133/gHTA and gPEHM, respectively) relatively to HERVs/MaLRs (3.5 probes/probeset on average, Fig. 2b, c), comparisons were carried out after stratification by intensity and probeset size. The low intensities observed in HERVs/MaLRs elements (Fig. 2a) are due to the fact that after embryonic development, a majority of retroelements are permanently repressed [28]. The reduced probesets size can, on the other hand, be attributed to the lack of large specific regions in HERVs/MaLRs loci that could allow the definition of bigger probesets.

gPEHM probesets were consequently regenerated such that the probeset size distribution in this new compartment, named “downsized gPEHM” (dgPEHM), matches this in HERVs/MaLRs. Repeatability and accuracy statistics were then computed. For a given intensity bin, the CVs were similar between gPEHM and gU133/gHTA, and dgPEHM and HERVs/MaLRs (see Fig. 2d), indicating that, after controlling for the confounding factors, the repeatability is similar across genomic elements and design methods. Nevertheless for a given intensity interval, HERVs/MaLRs and dgPEHM median CVs were approximately twice as large as gPEHM and gU133/gHTA due to probeset size heterogeneity. A similar trend was observed with the titration response curves (see Fig. 2e) built using probesets in the intensity bin]6; 15] : gPEHM and gU133/gHTA probesets reached the *y* = 100% asymptote at lower A/B and B/A ratios than HERVs/MaLRs and dgPEHM. More precisely, the ratio at which 75% of the probesets titrate is attained at *A*_*i*_/*B*_*i*_ = 1.43 and 1.52 in HERVs/MaLRs and dgPEHM, whereas the same ratio was reached at 1.23 and 1.24 in gPEHM and gU133/gHTA. The evaluation of accuracy using the titration mixtures led to a different trend, the theoretical values being *β*_C_ = 0.25 and *β*_D_ = 0.75. While *β*_C_ was better estimated in HERVs/MaLRs compartments (median ${\widehat{\beta}}_{\mathrm{C}}=0.30$) than in genes compartments (median ${\widehat{\beta}}_{\mathrm{C}}=0.40$), the opposite was observed with D (median ${\widehat{\beta}}_{\mathrm{D}}=0.78$ as compared to 0.59 in HERVs/MaLRs).

Overall, the observed differences in repeatability and titration response can essentially be attributed to the probeset size (restricted in HERVs/MaLRs owing to their repetitive nature) and not to the design method. The close examination of these results show that above a background noise of 2^6^, the performances do not differ substantially between HERVs/MaLRs and gU133/gHTA, where the median CV is 4 and 2% respectively. Relating these performances to the probeset size, we can conclude that, in comparison with gHTA/gU133 probesets populated by 10 probes on average, (i) gPEHM show nearly identical performances while having an average probeset size of 5.8 probes, and (ii) HERVs/MaLRs have comparable performances with an average probeset size of 3.5 probes. These results are in line with Lu et al. [58] who estimated that probesets should contain at least 4 probes for reliable interpretation.

### Consistency with Affymetrix design and model validation

**Fig. 3:**
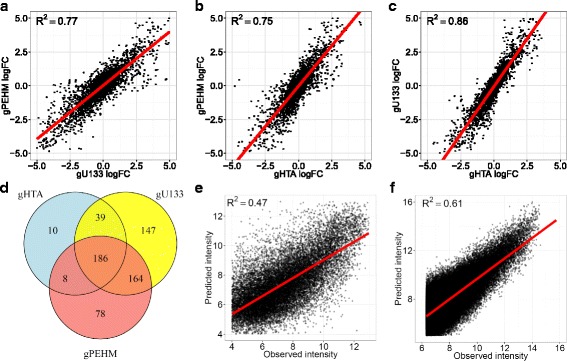
Consistency with Affymetrix design and model validation. Gene expression variation is compared across the three gene compartments based on fold-change correlation (**a**–**c**) and intersections of genes differentially expressed in the gene repertoires (**d**). The hybridization model PEHM is evaluated by correlating predicted and observed intensities on gU133 probes (**e**) and HERV-V2 training set (**f**)

Microarrays are generally used to measure the variation of transcript levels across two or more samples of interest. To assess the differential expression concordance among the gene repertoires, fold-changes and differentially expressed genes (DEG) were compared across the three gene repertoires. The log fold-changes between samples A and B were measured in the three gene compartments, leading to large *R* ^2^ values (see Fig. 3a–c). Although a higher correlation was obtained between the two Affymetrix repertoires (*R* ^2^ = 0.86), gPEHM showed a good coherence with Affymetrix fold changes (*R* ^2^ = 0.75, 0.77). These values are remarkably high given that gU133 and gPEHM probesets target genes 3′ UTR whereas gHTA covers all exons. Similarly, for a given repertoire, a large proportion of DEG are shared with the two others, these fractions being of 82.1, 75.4 and 95.9% in gPEHM, gU133 and gHTA respectively (Fig. 3b). Taken together, these results point toward a good concordance between Affymetrix and gPEHM probesets in the measure of gene expression variation, the smaller correlation with gPEHM being probably attributable to smaller probesets size in this compartment.

The last step in the platform evaluation consisted in the validation of PEHM. To this end, predicted intensities were generated from PEHM affinities and compared with those observed on the gU133 repertoire. For each gU133 probeset, the expression level was first estimated on two-third of the probes by regressing intensities onto PEHM affinities. Then, intensities were predicted on the last third of the probes by taking the product of PEHM affinities with the estimated expression level, leading to a *R* ^2^ = 0.47 between observed and predicted intensities (Fig. 3e). Although 0.14 lower than what was obtained on HERV-V2 (*R* ^2^ = 0.61, Fig. 3f), this value reflects a good ability of PEHM to model the probe-target affinity on HERV-V3, the discrepancy being probably due to the format change between HERV-V2 (11 micron, from which the model is trained), and HERV-V3 (5 micron) arrays.

When comparing the performances of PEHM (*R* ^2^ = 0.61) with the models proposed by Zhang et al. [40] (*R* ^2^ = 0.98) and Mei et al. [37] (*R* ^2^ = 0.82), our model may appears less predictive. This discrepancy probably reflects the differences in training set size (e.g. Zhang’s model) and in whether the RNA abundance is accounted for (e.g. Mei’s model). More precisely, while PEHM was evaluated on HERV-V2 training set consisting of 37,200 probes using total RNA from 15 different biological conditions, Zhang’s model was evaluated on 14 probesets whose targets were spiked at 14 varying concentrations, Mei’s model was, on the other hand, evaluated on all 25-mer probes spanning 90 human transcripts whose targets were spiked at 16 concentrations. Since their model was fitted for each concentration at a time, no abundance term θ was included. Of note, when testing Zhang’s model and Mei’s modified model (with the RNA abundance term θ added) on HERV-V2 training set, the performances found were R^2^ = 0.46 and 0.54, respectively, that is 0.15 and 0.07 less than PEHM performance (R^2^ = 0.61).

### Validation on characterized HERV loci

**Fig. 4:**
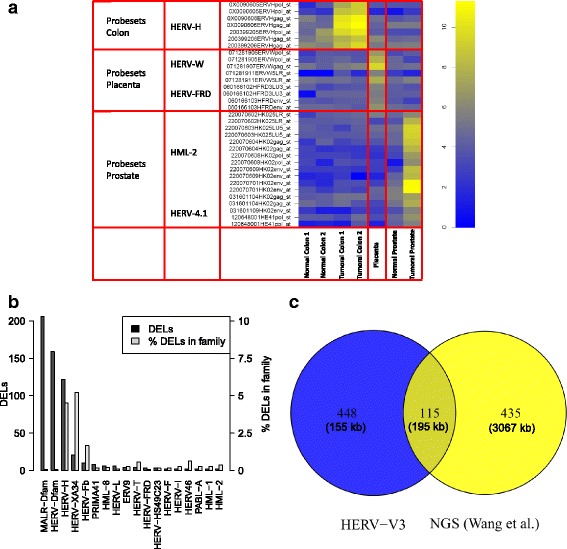
Biological validation. **a** Intensity heatmap of tissue and pathology specific loci in seven HERV-V3 arrays: the observed intensities correlate well with the expected loci specificity. For each of the eight locus, the family and the probesets names are indicated (the family name and the sub-region annotation are abbreviated in the probeset name). **b** Distribution of differentially expressed loci (DELs) between hPSCs and embryoid bodies. While most of LDEs are found in MaLR-Dfam, HERV-Dfam and HERV-H, when normalized within family, the proportion of LDEs is higher in HERV-H and HERV-XA34, consistently with Wang et al. [13]. **c** Intersection between pluripotent loci identified by HERV-V3 and NGS (Wang et al.): despite a small number of shared loci (115), 55.7% of HERV-V3 loci coverage is contained in this intersection

Previous studies have revealed that certain HERV loci are expressed in a tissue, pathology and developmental stage specific manner and can potentially be used as biomarkers. In a perspective of biological validation, we sought to replicate these results on HERV-V3. We first evaluated whether HERV loci previously characterized by RT-PCR in placenta [29, 59, 60], prostate [61], Cheynet et al. unpublished data and colon tumor [6], showed similar expression patterns on HERV-V3. The heatmap Fig. 4a indicates that the intensities observed on the array are consistent with the expected patterns of expression: cancer and tissue specific loci are transcriptionally active only in their associated sample. The expression and tissue tropism of those loci were subsequently confirmed by RT-PCR (Additional file 6: Figure S3), with the same biological samples used in the microarray experiments. Cross-reactions were checked on the same loci by examining probesets targeting their closest paralogous sequences using blat [62]. For these probesets at risk of cross-hybridization, the intensity was under the background noise, pointing toward a high level of specificity of the array (Additional file 7: Figure S4).

Other works have shown the involvement of HERV-H in the maintenance of pluripotency, among which Wang et al. who found 550 HERV-H copies transcribed at higher level in human pluripotent stem cells (hPSCs) compared with embryoid bodies (early stage of hPSCs differentiation) [13]. To determine whether a similar enrichment in HERV-H elements was also found on HERV-V3, we searched for differentially expressed loci (DELs) between OSCAR and EBJ14, two primary human cell lines with differentiation levels similar to those in Wang et al. 563 loci were identified as differentially expressed, among which 122 belong to HERV-H family (see Fig. 4b). Given that HERV-H represents only 0.4% of the probesets on HERV-V3, this high proportion (21.7%) of HERV-H in the set of DELs argues in favor of non-random expression of HERV families (binomial test, *p* < 2.10^-16^) and confirms the trend observed in NGS studies. It can be noted that the majority of the DELs are MaLRs, which is in line with Fort et al. who also observed the reactivation of these elements in human embryonic stem cells, although to a smaller extent than in mouse [24]. Finally, DELs positions were intersected with Wang loci, leading to 115 common regions spanning a total of 195 kb (Fig. 4c). While modest, this intersection represents 55.7% of the total DELs coverage and cannot be attributed to chance (binomial test, *p* < 2.10^-16^). The discrepancy with Wang et al. is likely due to differences in sample (different cell lines) and assay (NGS versus microarray). Nevertheless, three HERV-H loci and one MaLR element identified as OSCAR specific on the microarray were validated by RT-PCR (Additional file 6: Figure S3), confirming thus the observed pattern on HERV-V3.

## Conclusions

The recent development of high-throughput genomic approaches has enabled biologists to perform global analysis of gene expression. These technological advances have made possible to investigate disease mechanisms, identify biomarkers [63], group genes into functional pathways [64], assign function to previously unannotated genes, and evaluate the toxicity of candidate drugs [65]. Among those technologies, microarrays have been widely utilized in clinical studies for their cost-effectiveness, their rapid and mature turnaround, and their ability to provide high sensitivity and specificity results from limited biological materials (nanograms). In this work, we have presented a new high-density array allowing the examination of the whole HERVs/MaLRs transcriptome along with a selection of genes, LINE-1 elements and exogenous viruses. Such configuration opens the door to multiple applications such as the identification of enhancers and alternative promoters, the simultaneous detection of viruses and monitoring of genes and HERVs/MaLRs modulation, the identification of new biomarkers, etc. This was made possible by the careful detection and annotation of HERVs/MaLRs as well as the development of PEHM, allowing the optimization of probe performances and the control of cross-reactions. The evaluation of the platform showed that, (i) after controlling for confounding variables, similar levels of reproducibility and accuracy were obtained between Affymetrix and HERV-V3 arrays; (ii) a high consistency was found between gU133, gHTA and gPEHM probesets in term of GDE detection; (iii) for a selection of tissue/pathological loci specific, the pattern of expression reported in the literature was also observed on HERV-V3. In 2008, Mayer et al. highlighted the need for a HERV transcriptome project to study the contribution of HERVs as part of the human transcriptome [66]. Although previous works measured individual HERVs expression on a limited scale [6, 23], to our knowledge no such project has been setup yet, probably due to the technical difficulties inherent to REs. Because of its performances and exhaustiveness, HERV-V3 could benefit such project.

## Additional files

Additional file 1: Supplementary notes. (DOCX 22 kb)

Additional file 2: Table S1. Chromosome locations of the prototypes used in HERVgDB4 generation. For each of the 70 prototypes associated with 42 HERV families, the family name, the sub-region annotation (full length provirus, int = gag + pol + env, LTRs, U3, R, U5 subdomains, and gag, pol, dUTPase, env genes), chromosome location (chromosome, start, end) and strand are provided. The 42 HERV families split into, 28 class I, 11 class II and 3 class III sub-families. (XLS 76 kb)

Additional file 3: Figure S1. Models performance illustrated on gene CD59. (PDF 223 kb)

Additional file 4: Table S2. List of the 1559 genes used for the PEHM hybridization model evaluation. For each gene, abbreviated name, full name, alias and accession number are provided. As indicated in the paper, each of these genes is targeted by three probesets, two derived from Affymetrix arrays U133 (GeneChip Human Genome U133 Plus 2.0 Array), HTA (GeneChip Human Transcriptome Array 2.0) and one designed using our probes and probesets selection procedure. (XLS 119 kb)

Additional file 5: Figure S2. Correlation between gU133 probesets on HG-U133 Plus 2.0 and HERV-V3 microarrays. (PDF 281 kb)

Additional file 6: Figure S3. RT-PCR validation on loci specific of placenta, colon and prostate tumor tissues, and, embryonic stem cells. (PDF 357 kb)

Additional file 7: Figure S4. HERV-V3 specificity evaluation. (PDF 315 kb)

## Abbreviations

DEG: Differentially expressed gene
DEL: Differentially expressed locus
dgPEHM: downsized gPEHM
EST: Expressed sequence tag
gU133, gHTA, gPEHM: Probesets that originate from commercial Affymetrix arrays (U133 and HTA v2) and our design. They target 1559 genes involved in eight pathways (immunity, inflammation, cancer, central nervous system affections, differentiation, telomere maintenance, chromatin structure and gag-like genes, see Additional file 4: Table S2)
HERV: Human endogenous retrovirus
hESCs: human stem cells
lincRNA: long intergenic non-coding RNA
lncRNA: long non-coding RNA
LTR: Long terminal repeat
MaLR: Mammalian apparent LTR-retrotransposon
MM: Mismatch probe
PEHM: Pentamer rEgion-dependent Hybridization Model
PM: Perfect match probe
RE: Repetitive elements
TSS: Transcription start site
