# Supplementary material for: A comprehensive hybridization model allows whole HERV transcriptome profiling using high density microarray
Source: BMC Genomics. 2017 Apr 8;18:286. doi: 10.1186/s12864-017-3669-7 (PMC5385096; doi:10.1186/s12864-017-3669-7)
Supplement: Supplementary file 1 — Supplementary notes. (DOCX 22 kb) [file 12864_2017_3669_MOESM1_ESM.docx]

Additional file 1

A Comprehensive Hybridization Model Allows Whole HERV Transcriptome Profiling Using High Density Microarray
SUPPLEMENTARY NOTES

*Jeremie Becker¹, Philippe Perot¹ , Valerie Cheynet¹, Guy Oriol¹, Nathalie Mugnier², Jean Baptiste Veyrieras² and Francois Mallet¹ .*

*¹ : Medical Diagnostics Discovery Department, bioMerieux, 376 Chemin de l'Orme, 69280 Marcy l'Etoile, France.*

*² : Bioinformatics Research Department, bioMerieux, 376 Chemin de l'Orme, 69280 Marcy l'Etoile, France.*

Contents

This document contains supplementary notes and figures referred to in the main text.

1 Prototypes definition.

2 Database creation.

3 Supplementary tables and figures legends.

4 References.

1 Prototypes definition

**Rational**

The rational for designing HERV prototype sequences is based on an expected added value in comparison to consensus sequences, those prototypes allowing both the identification of HERV copies on the human genome and their optimal functional annotation into subregions, U3, R and U5 within LTRs, gag, pol, env (rec, dUTPase) in internal regions. Thus, to get the most comprehensive annotations, the ideal prototype would be the proviral sequence corresponding to the ancestral infectious retrovirus. In practice, for each family described in the literature, we sought to identify the most conserved sequence, i.e., the one which maintains the largest open reading frames for gag pol env genes within a proviral structure flanked by two complete LTRs sequences., For divergent families, several prototypes associated with different loci were used to improve sensitivity and provide comprehensive annotation.

**Prototypes curration**

Prototype sequences were named according to Medstrand and Mager (2003), and reference clones were selected from the literature (Seifarth et al., 2003, Frank et al., 2005, Jern et al., 2005, Strissel et al., 2012, Perot et al., 2012). In the absence of a reference sequence that met all our criteria for a prototype and/or to complement the search for an almost functional sequence within a family, the Dfam database (Wheeler et al., 2013), based on Repbase consensus, was used to select the longest provirus exhibiting the canonical 5'LTR / gag / pol / env / 3'LTR structure. Few prototypes (eg HERV-I) were also selected from Repbase (Jurka et al., 2005) which contains prototype and not consensus sequences for a small number of families (<5%). Lastly, when absent from Dfam (and Repbase), the Retrovirus Reference Sequence Library documented online (Paleovirology web site by Gifford et al., 2013) was used as a supplementary source of prototype sequences (eg HML9). Altogether, 70 annotated prototypes covered 42 families, i.e. 47, 19 and 4 loci targeting 28 class I, 11 class II and 3 class III families, respectively. Self dotplot analyses were performed to define LTRs boundaries. The definition of LTRs U3, R and U5 subdomains was obtained using online softwares.

| Domains | Function | Name | URL |
| --- | --- | --- | --- |
| LTR | Identification of LTR and PBS/PPT | LTR finder | <http://tlife.fudan.edu.cn/ltr_finder/> |
| U3 | transcription factors and pol II sites | Promoter scan | <http://www-bimas.cit.nih.gov/molbio/proscan/> |
| U3 | Promotors and TATAA boxes | Softberry_FPROM | <http://linux1.softberry.com/berry.phtml?topic=fprom&group=programs&subgroup=promoter> |
| U3 | transcription factors, TBP, NFY | Consite | <http://asp.ii.uib.no:8090/cgi-bin/CONSITE/consite?rm=t_input_single> |
| U3 | TATAA box, CAAT box et GC box | Gene Promoter Miner | <http://gpminer.mbc.nctu.edu.tw/index.php> |
| U3 | facteurs de transcription | Signal Scan | <http://www-bimas.cit.nih.gov/molbio/signal/> |
| R | Start of transcription | TSSG /TSSW | <http://linux1.softberry.com/berry.phtml?topic=tssg&group=programs&subgroup=promoter> <http://linux1.softberry.com/berry.phtml?topic=tssw&group=programs&subgroup=promoter> |
| R | polyA prediction | Softberry_polyah | <http://linux1.softberry.com/berry.phtml?topic=polyah&group=programs&subgroup=promoter> |
| R | polyA prediction | polyA SVM | <http://exon.umdnj.edu/polya_svm_server/index.html> |
| R | EST analysis | BLAST NCBI | http://blast.ncbi.nlm.nih.gov/Blast.cgi |

**Prototypes annotation and refinement**

For each of the HERV prototype, a search of ORF (gag, pol, env) was performed using Retrotector (Sperber et al., 2009). dUTPase sites were identified in all HML prototypes using the HML-2 HOM dUTPase sequence as reference (Mayer and Meese , 2003). In order to refine Retrotector annotations, especially to better define the 5' and 3' ends of genes, proteins from 70 infectious retroviruses (alpha-, beta-, gamma-, epsilon-, delta-, spuma-like, and lenti- retroviruses) were gathered and aligned on the prototypes using fasty36 (http://fasta.bioch.virginia.edu). ORF boundaries were finally verified using the ORF prediction tool of Geneious 7.1.4. (Kearse et al., 2012).In addition to LTRs and ORFs annotations, potentially donor or acceptor splicing sites were sought using two online softwares Human Splicing finder (http://www.umd.be/HSF/) and Berkeley Drosophila Genome Project (<http://www.fruitfly.org/seq_tools/splice.html>).

All the prototypes can be found in supplementary table 1. For the detection step, the prototypes were both used as a whole and at the sub-region level to identify HERVs in their heterogeneous forms (from intact to highly fragmented and degenerated). For the annotation step, only the sub-region prototypes were utilized, i.e. U3, R and U5 for LTRs and gag, dUTPase, pro, pol, env for internal regions.

2 Database creation

**HERV reconstruction**

After (i) running RepeatMasker using our prototypes and (ii) collecting predictions from Dfam, a post-processing step was necessary to turn hits into HERV copies. Indeed, multiple hits (predictions) can originate from a single copy due to the divergence between the copy and the prototypes (or HMM profiles), as well as large insertions deletions events. A parser was thus implemented to (i) remove redundancy across predictions, (ii) merge nearby or overlapping predictions and (iii) group closeby LTRs and internal regions in a single copy. Because the two methods provided overlapping predictions, the priority was given to the well-annotated HERV prototypes by removing HERVs Dfam elements intersecting HERV prototypes.

**Annotations**

While the detection step allows to determine which family an element belongs to, the annotation step enables the identification of functional regions within a particular element. These annotations allow (such as promoter or polyA), splicing strategies (based on predicted SD/SA sites) and other motives (putative ORFs, transcription factor binding sites, deletion patterns) when relevant.

HERV prototypes and LINE-1 were annotated by aligning them against sub-regions prototypes (U3, R, U5, ORFs) using RepeatMasker. A step of parsing (as described above) was also needed to reconstruct the fragmented predictions generated by RepeatMasker. The level of annotation in RepBase only allow to distinguish LTRs from internal regions, consequently, HERVs Dfam (detected with RepBase consensus) carry this level of annotation.

3 Supplementary Tables and figures legends

**Supplementary table 1**. chromosome locations of the prototypes used in HERVgDB4 generation.

For each of the 70 prototypes associated with 42 HERV families, the family name, the sub-region annotation (full length provirus, int =gag+pol+env, LTRs, U3, R, U5 subdomains, and gag, pol, dUTPase, env genes), chromosome location (chromosome, start, end) and strand are provided. The 42 HERV families split into, 28 class I, 11 class II and 3 class III sub-families.

**Supplementary table 2**. List of the 1519 genes used for the PEHM hybridization model evaluation.

For each gene, abbreviated name, full name, alias and accession number are provided. As indicated in the paper, each of these genes is targeted by three probesets, two derived from Affymetrix arrays U133 (GeneChip® Human Genome U133 Plus 2.0 Array), HTA (GeneChip® Human Transcriptome Array 2.0) and one designed using our probes and probesets selection procedure.

**Supplementary figure 1**. Model selection illustrated on CD59 gene.

The development of PEHM involved a step of model selection. To do so, 14 models accounting for various k-mer size (k є [2,5], rows) and spatial features (columns) were fitted on HERV-V2 (the previous generation of HERV array). We recall that the originality of our approach lies in the explicit modeling of MMs and Gaps in addition to PMs. As presented section 3, two strategies have been proposed in the literature to account for the spatial effect in probes, either through position weights (Zhang et al., 2003) or by estimating k-mers at each position of the probe (Mei et al., 2003). Two alternatives were also included in the comparison, an approximation of the latter consisting in splitting probes into three sub-regions, and, models without spatial information. Empty boxes correspond to combinations of k-mers and spatial modeling approaches that required an excessive number of parameters and could consequently not be run on our servers.

Each line corresponds to a probeset measure in a microarray, the colors reflect the target concentration (yellow for high, red for low). Out of 11 probes contained in the CD59 probeset, 9 are represented, the two others being filtered out because of cross-reaction risk. Similarly, certain probes in particular arrays were flagged as outliers when fitting the model, and consequently excluded. On the right panel, the observed intensities are represented, revealing the actual “affinity footprint” of the probeset : probes 2,5,8 and 9 have higher affinity than probes 3,6 and 7. Two trends appear on the figure : predictions are improved when k-mers size increases and when spatial information modeling is finer. In this way, the 3 regions, 5-mers model (PEHM) accurately predicts the affinity variations in CD59 probeset. By contrast, models with smaller k-mers give a rough approximation of affinity, where only the affinity drop at probe 6 seems to be properly captured.

4 References

Frank O, Giehl M, Zheng C, Hehlmann R, Leib-Mösch C 1 Seifarth W. Human endogenous retrovirus expression profiles in samples from brains of patients with schizophrenia and bipolar disorders. *J Virol.* **79**, 10890-10901 (2005).

Gifford RJ, <http://saturn.adarc.org/paleo> (2013).

Jern P, Sperber GO, & Blomberg J. Use of endogenous retroviral sequences (ERVs) and structural markers for retroviral phylogenetic inference and taxonomy. *Retrovirology* **2**:50 (2005).

Jurka J, Kapitonov VV, Pavlicek A, Klonowski P, Kohany O & Walichiewicz J. Repbase Update, a database of eukaryotic repetitive elements. *Cytogenetic and Genome Research* **110**, 462-467 (2005).

Kearse M, Moir R, Wilson A, Stones-Havas S, Cheung M, Sturrock S, Buxton S, Cooper A, Markowitz S, Duran C, Thierer T, Ashton B, Mentjies P & Drummond A. Geneious Basic: an integrated and extendable desktop software platform for the organization and analysis of sequence data. *Bioinformatics* **28**, 1647-1649 (2012).

Mager DL & Medstrand P. Retroviral repeat sequences, In *Nature encyclopedia of the human genome*. Edited by Cooper D, Nature Publishing Group, London, United Kingdom **5**, 57-63. (2003).

Mayer J & Meese EU. Presence of dUTPase in the various human endogenous retrovirus K (HERV-K) families. *J Mol Evol.* **57**, 642-9 (2003).

Seifarth W, Spiess B, Zeilfelder U, Speth C, Hehlmann R, & Leib-Mösch C. Assessment of retroviral activity using a universal retrovirus chip. *J Virol Methods*. **112**, 79-91 (2003).

Sperber G, Lövgren A, Eriksson NE, Benachenhou F & Blomberg J. RetroTector online, a rational tool for analysis of retroviral elements in small and medium size vertebrate genomic sequences. BMC *Bioinformatics* **16**, Suppl 6:S4. (2009).

Strissel PL, Ruebner M, Thiel F, Wachter D, Ekici AB, Wolf F, Thieme F, Ruprecht K, Beckmann MW, & Strick R. Reactivation of codogenic endogenous retroviral (ERV) envelope genes in human endometrial carcinoma and prestages: Emergence of new molecular targets. *Oncotarget*. **3**, 1204-1219 (2012).
