# Supplementary material for: A comprehensive hybridization model allows whole HERV transcriptome profiling using high density microarray
Source: BMC Genomics. 2017 Apr 8;18:286. doi: 10.1186/s12864-017-3669-7 (PMC5385096; doi:10.1186/s12864-017-3669-7)
Supplement: Supplementary file 3 — Models performance illustrated on gene CD59. (PDF 223 kb) [file 12864_2017_3669_MOESM3_ESM.pdf]

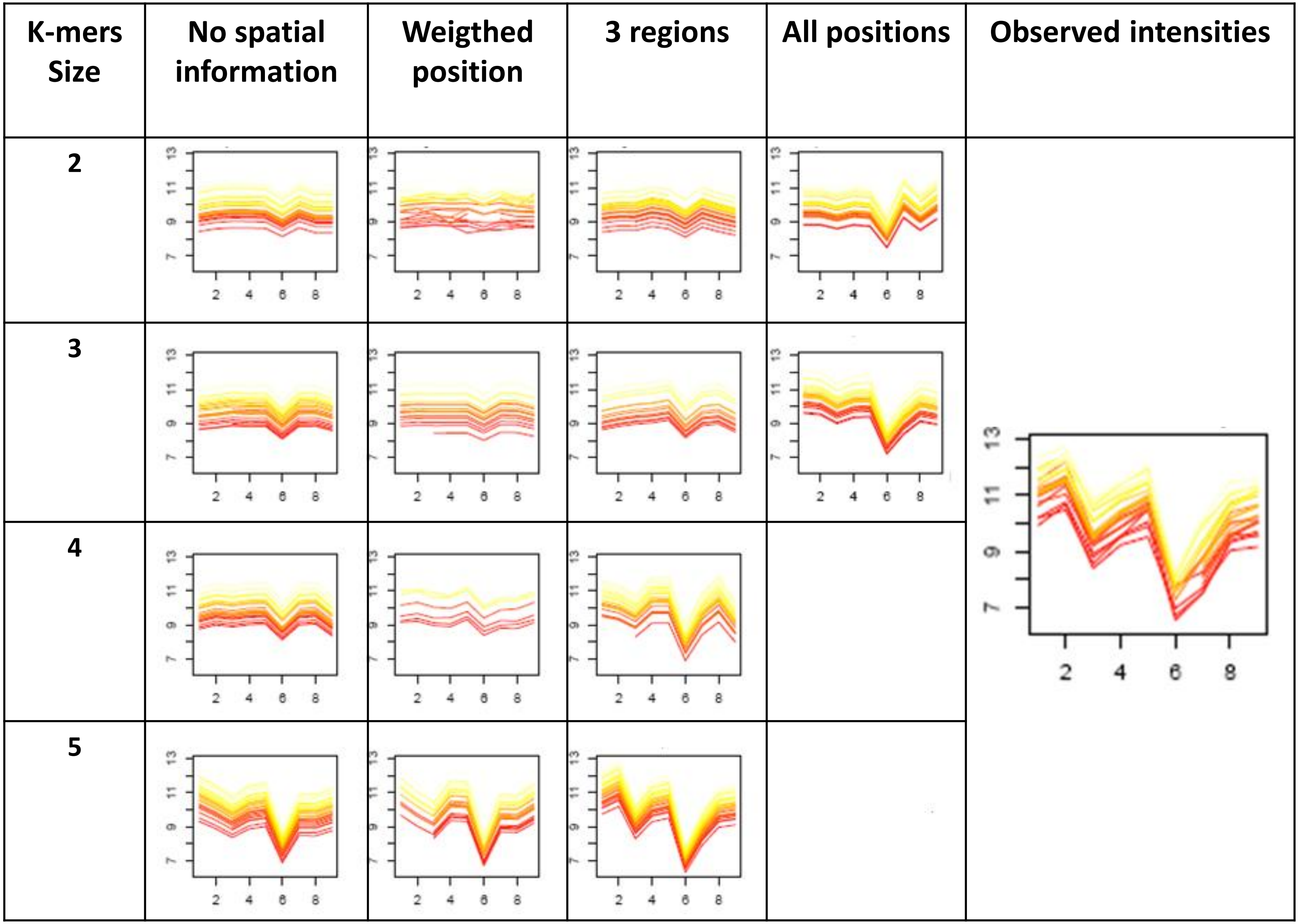

Supplementary figure 1 : Models performance illustrated on CD59 gene. 14 models were evaluated before defining PEHM by combining the k-mers size and various way to account for spatial information. The larger the k-mers are as well as the more elaborated the spatial information is, the better the models fit the observed intensities.
