# Supplementary material for: A comprehensive hybridization model allows whole HERV transcriptome profiling using high density microarray
Source: BMC Genomics. 2017 Apr 8;18:286. doi: 10.1186/s12864-017-3669-7 (PMC5385096; doi:10.1186/s12864-017-3669-7)
Supplement: Supplementary file 5 — Correlation between gU133 probesets on HG-U133 Plus 2.0 and HERV-V3 microarrays. (PDF 281 kb) [file 12864_2017_3669_MOESM5_ESM.pdf]

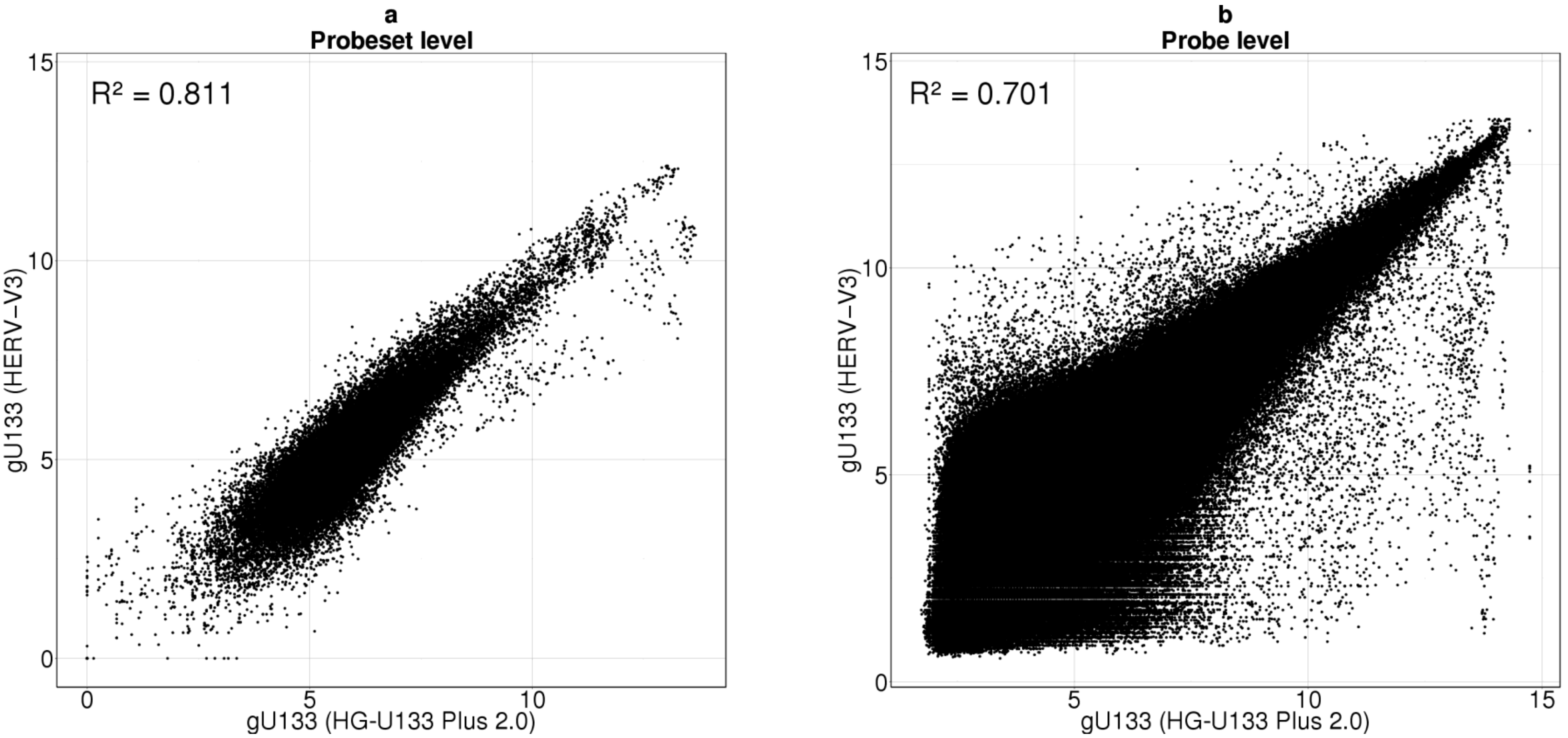

Supplementary figure 2 : Correlation between gU133 probesets on HG-U133 Plus 2.0 and HERV-V3 microarrays. Correlations were computed on the probeset **(a)** and probe **(b)** levels. The data were generated from the same four MAQC samples used in the Platform Evaluation section, where each of them was performed in technical triplicate. All protocols were identical (see Materials and Methods), except for HG-U133 Plus 2.0 where only 5µg of dsDNA were hybridized according to Affymetrix guidelines After quality check, the data were pre-processed using RMA-TRPN normalization.
