# Supplementary material for: A comprehensive hybridization model allows whole HERV transcriptome profiling using high density microarray
Source: BMC Genomics. 2017 Apr 8;18:286. doi: 10.1186/s12864-017-3669-7 (PMC5385096; doi:10.1186/s12864-017-3669-7)
Supplement: Supplementary file 6 — RT-PCR validation on loci specific of placenta, colon and prostate tumor tissues, and, embryonic stem cells. (PDF 357 kb) [file 12864_2017_3669_MOESM6_ESM.pdf]

RT-PCR validation of HERV loci that are specific of placenta, colon and prostate tumor tissues, and, embryonic stem cells.

RT-PCRs were performed on 5 tissues and cell lines (placenta, prostate, colon, OSCAR and EBJ14) as follow. Total RNA (200 ng) was DNase-treated and reverse transcribed (QuantiTec Reverse Transcription Kit, Qiagen) in 20 µL reaction volume. SYBR green experiments were set up using the Type-it HRM PCR kit in 20 µL final reaction volume with 0.3 µM primers and 9 µL of 20-fold cDNA dilution. The cDNA amplification were performed as follows: a 5 min denaturation step at 95°C followed by 40 cycles (95°C for 10s, Tm for 30s, 72°C for 10s) and melting curve analysis. TaqMan experiments were performed in a volume of 20 µL containing 1 µL TaqMan® Gene Expression Assay 20X, 10 µL TaqMan® 2X Universal PCR Master Mix, No Amperase® UNG (Applied Biosystems) and 9 µL of 20-fold diluted cDNA. Thermal cycling conditions were: 10 min at 95 C, followed by 40 cycles of amplification with 15 s at 95 C and 1 min at 60 C. All reactions were performed in duplicates. Expression of housekeeping genes G6PD, RPLPO and GAPDH was monitored for normalization. Relative Expression (RE) values were defined as EffΔCt, in which ΔCt = Ctmin-series – Ctsample. Loci of interest values were normalized by using the geometric mean of RE values corresponding to G6PD, RPLPO and GAPDH. Within a sample series, the lowest normalized RE value was finally arbitrary set at one and other values scaled up in order to provide a final relative differential expression view. Graphical representations were generated using GraphPad Prism 5.0.

PCR Primer Sequences

Locus	Forward primer	Reverse primer
HERV-W / **07**12819 (Syncytin1 / ERVWE1)	TaqMan assay HS02341206_g1 (Applied Biosystems)	
HERV-FRD / **06**01661 (Syncytin2 / ERVFRDE1)	TaqMan assay HS01652148_m1* (Applied Biosystems)	
HERV-H / **0X**00906	CAGGCGTTGCTGAGTGTGTCTAATC	TGGAGCCTGAGGAAGAATTGGGACC
HERV-H / **20**03992	CCCAAGCGGCGCTGAGTCTT	TGGGATGAAGGGAGGGGAGGC
HML-2 / **22**00706	TTCCACCTGAGGAGAAATGC	GACCACACTCGAGAGTCCCT
HERV-H / **04**30884	AGTGAGTTGTGGAGGAGGGTAT	TCGCCTGCTACAAAATTGCC
HERV-H / **03**08707	GAAACGTCTGGCTGCTACAGT	TCACCTTCAAGGTGTGAAATAACAG
HERV-H / **04**00516	ACCTCCCCCACTGAGTACAT	GGGGCCGTTTTATAGGATTTCG
**08**1709501-MALR1007uL_at	TATTATGTCAAATCTTTCATGCAC	TCATACAGGCCCTTTAAATCT

HERV-W (Syncytin-1) and HERV-FRD (Syncytin-2) loci expressed only in the placenta tissue.

Family / locus name (this work). Chromosomal localization	Expression detected using HERV-V3 and previous generation microarrays		RT-PCR validation
	Arrays	Probesets	
HERV-W / **07**12819<br>chr7:92467962-92478192<br>(Syncytin1 / ERVWE1)	HERV-V3	071281911ERVW5LR<br>071281907ERVWgag<br>071281905ERVWpol	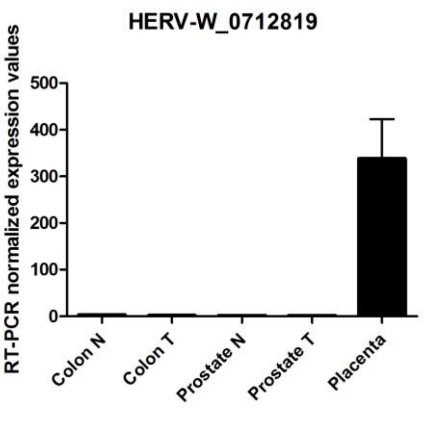
	HERV-V2	700341_w_L5R<br>700341_w_gag<br>700341_w_pol	
	HERV-V1	HW28073.L5.U5<br>envw_at<br>sp.ervwe1_at	
HERV-FRD / **06**01661<br>chr6:11102749-11112216<br>(Syncytin2 / ERVFRDE1)	HERV-V3	060166103HFRDenv<br>060166102HFRD3LU3	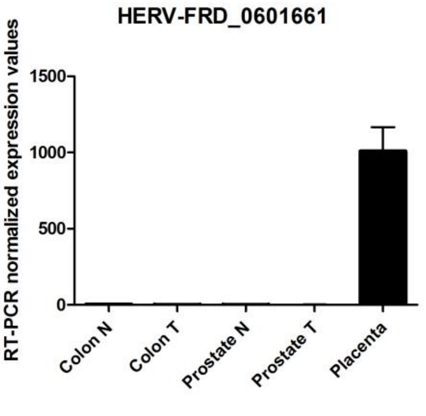
	HERV-V2	600084_f_env<br>600084_f_L3U3	
	HERV-V1	envfrd_at	

HERV-H and HML-2 loci expressed specifically in colon or prostate cancer tissues.

Family / locus name (this work). Chromosomal localization	Expression detected using HERV-V3 and previous generation microarrays		RT-PCR validation
	Arrays	Probesets	
HERV-H / **0X**00906<br>chrX:4540474-4546320	HERV-V3	0X0090605ERVHgag<br>0X0090606ERVHpol	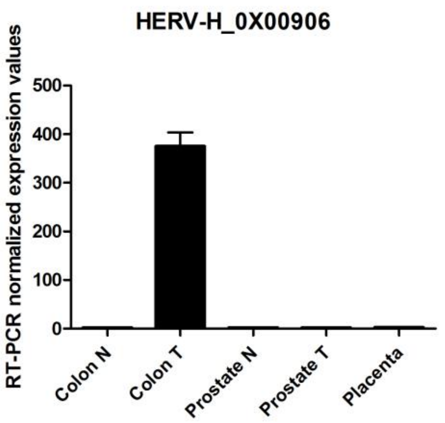
	HERV-V2	X00041_h_gag<br>X00041_h_pol	
HERV-H / **20**03992<br>chr20:19752047-19756776	HERV-V3	200399205ERVHgag<br>200399206ERVHpol	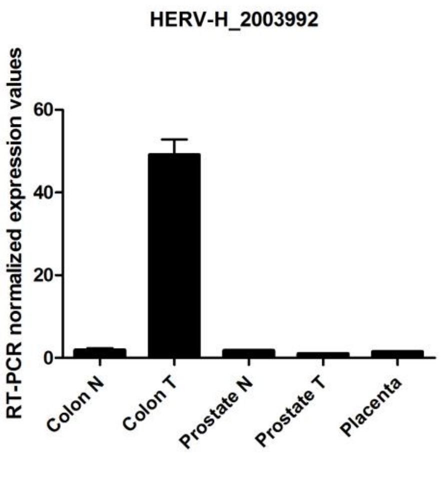
	HERV-V2	2000045_h_gag<br>2000045_h_pol	
HML-2 / **22**00706<br>chr22:23537740-23546008	HERV-V3	220070602HK025LR<br>220070603HK025LU5<br>220070604HK02gag<br>220070608HK02pol<br>220070609HK02env	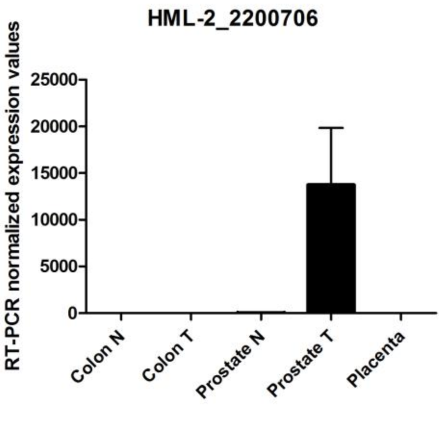
	HERV-V2	2200033_2_L5R<br>2200033_2_L5U5<br>2200033_2_gag<br>2200033_2_env	

HERV-H and MalR loci specifically expressed in human embryonic stem cells.

Family / locus name (this work). Chromosomal localization	Expression detected using HERV-V3 and previous generation microarrays		RT-PCR validation
	Arrays	Probesets	
HERV-H / **04**30884<br>chr4:179166474-179170568	HERV-V3	043088405ERVHpol_at	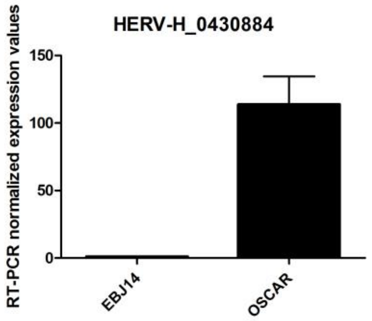
	HERV-V2	400831_h_gag_at	
HERV-H / **03**08707<br>chr3:54634482-54640204	HERV-V3	030870706ERVHgag_at	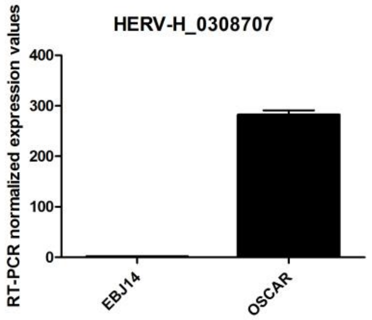
HERV-H / **04**00516<br>chr4:3927446-3933082	HERV-V3	040051607ERVH3LU3I_at	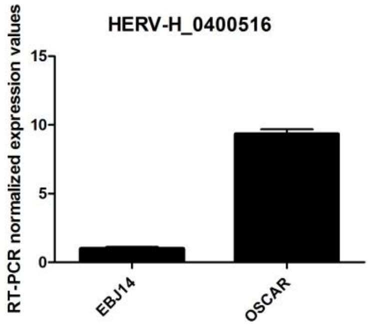
MALR1007 / **08**17095<br>chr8:114297028-114297284	HERV-V3	081709501-<br>MALR1007uL_at	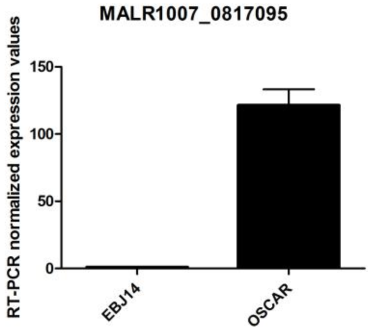
