# Supplementary material for: A comprehensive hybridization model allows whole HERV transcriptome profiling using high density microarray
Source: BMC Genomics. 2017 Apr 8;18:286. doi: 10.1186/s12864-017-3669-7 (PMC5385096; doi:10.1186/s12864-017-3669-7)
Supplement: Supplementary file 7 — HERV-V3 specificity evaluation. (PDF 315 kb) [file 12864_2017_3669_MOESM7_ESM.pdf]

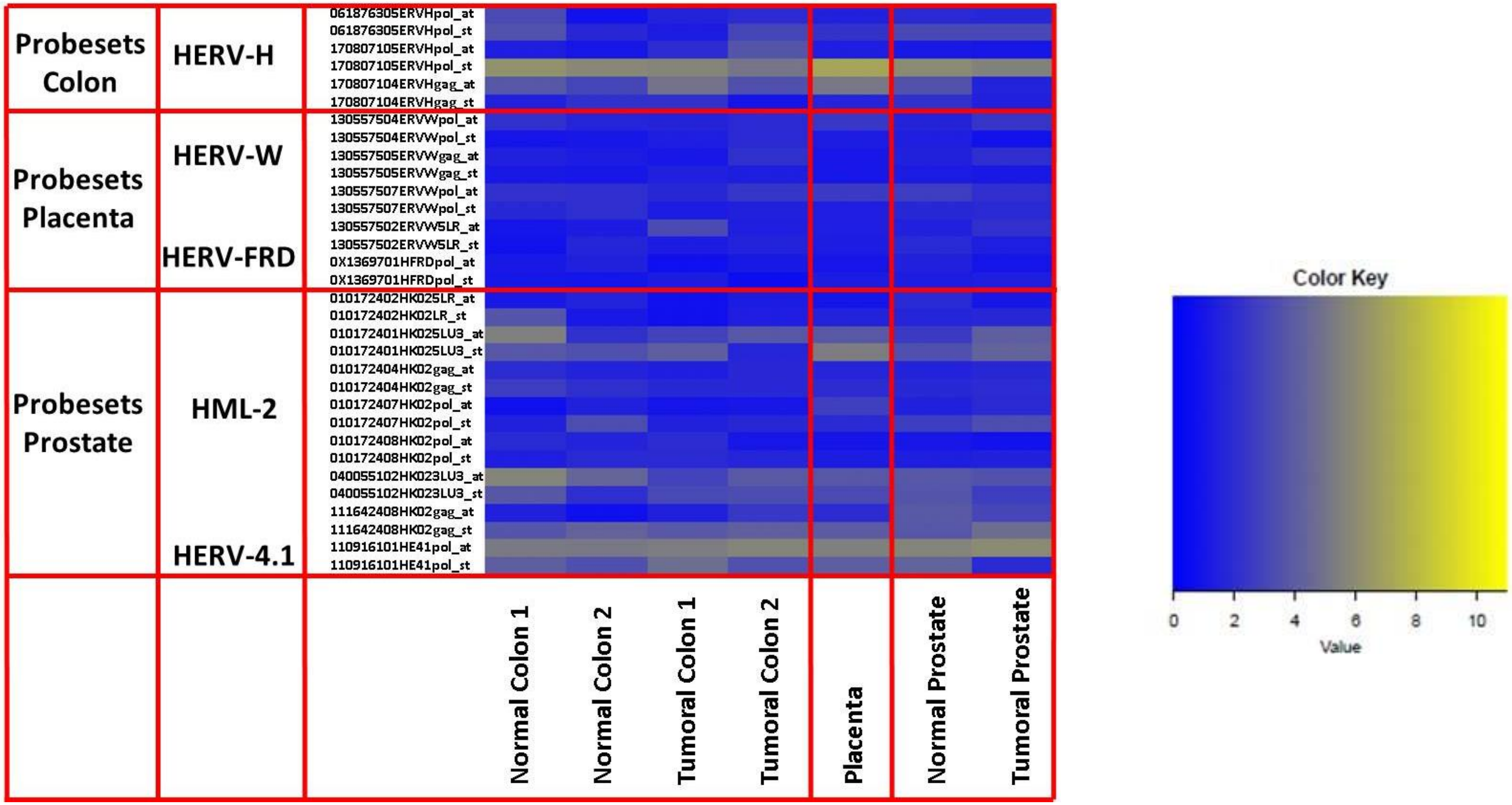

Supplementary figure 3 : To get a sense of HERV-V3 specificity, each locus used in the biological validation was aligned against hg19 using the online blat alignment tool. The best hit intersecting our database (HERVgDB4) was identified and its expression in the colon, placenta and prostate arrays was recorded (same samples as used in figure 4). When comparing the obtained heatmap with figure 4, it can be noticed that for most of these elements, the measured intensity is much lower than their counterpart used in the biological validation. Although this evaluation is limited to a small number of loci, it nevertheless suggests an absence of cross-reaction between a given target and non-specific probesets, associated with closely related targets.
